# Methyltransferase SETD7 as a Regulator of STING-Dependent Cytokine Response in Lung Cancer Cells

**DOI:** 10.3390/ijms27094020

**Published:** 2026-04-30

**Authors:** Ivan A. Nevzorov, Polina Korableva, Oleg Shuvalov, Sergey Parfenyev, Nickolai A. Barlev, Alexandra Daks

**Affiliations:** 1 Institute of Cytology, Russian Academy of Sciences, 194064 St. Petersburg, Russia; nevzorovia10@gmail.com (I.A.N.); korablevapolina936@gmail.com (P.K.); oleg8988@mail.ru (O.S.); gen21eration@gmail.com (S.P.); 2 Department of Biomedical Sciences, School of Medicine, Nazarbayev University, Astana 010000, Kazakhstan; 3 National Laboratory of Astana, Astana 010000, Kazakhstan

**Keywords:** SETD7, STING signaling, TBK1, IRF3, cytokines, type I interferon, innate immunity, apoptosis, salmon sperm DNA transfection

## Abstract

The innate immune signaling pathway cGAS–STING plays an important role in the recognition of cytosolic nucleic acids and the induction of the interferon-dependent antiviral response. Despite the significant research interest in this cascade in the context of immune system function, the mechanisms regulating cGAS–STING signaling and the switch between its pro-inflammatory and pro-apoptotic effects remain largely underexplored. According to publicly available RNA-seq data and microarray analyses, SETD7 lysine methyltransferase participates in interferon signaling in cancer cells. This study aims to elucidate the role of SETD7 in the regulation of the STING-dependent immune response in human lung adenocarcinoma (LUAD) cells. For this purpose, we developed a reproducible and cost-effective method for inducing the STING cascade by transfecting cells with salmon sperm DNA (sspDNA). We demonstrated that sspDNA efficiently induces phosphorylation of the key components of the STING–TBK1–IRF3 signaling pathway and activates the expression of interferons and pro-inflammatory cytokines. Using this approach, we further demonstrated that SETD7 is involved in the regulation of the IRF3-dependent transcriptional program. Suppression of SETD7 was associated with changes in the expression of genes related to innate immune response and apoptosis, including increased levels of *IFNA1*, *IL1B*, *BAK1*, *BBC3* (PUMA), and *BCL2*. Furthermore, attenuation of SETD7 expression reduced the lentiviral transduction efficacy in H1299 cells. These results suggest that SETD7 may play a role in regulating the switch in STING signaling between pro-inflammatory and pro-apoptotic responses in LUAD cells.

## 1. Introduction

Lysine-specific methyltransferase (KMT) SETD7 (Set7/9, KMT7) is known as a versatile epigenetic regulator that acts through promoting mono-methylation of lysine residues of both histone and non-histone targets [1]. Originally, SETD7 was described as a histone-specific enzyme promoting mono-methylation of lysine at position 4 in histone H3 (H3K4) [2]. This modification, in particular, in enhancer regions, is associated with chromatin relaxation and transcription enhancement [3]. It was later discovered that SETD7 also methylates histones H1, H1.4, H2A, and H2B; however, the effects of these modifications are not well established to date [4]. Importantly, SETD7 adds methyl groups to a large number of non-histone protein targets, including p53, NF-κB (p65/RelA), DNMT1, STAT3, β-catenin, E2F1, and others [5,6,7,8,9]. Affecting stability, activity, subcellular localization, and interactomes of transcription factors and key participants of cellular processes, SETD7 methyltransferase plays a multifaceted role in regulation of the cell cycle, stress response, DNA repair, and inflammatory reactions.

Given the wide range of SETD7 targets and its participation in key cancer-associated cellular processes, it is not surprising that the perturbed levels of this methyltransferase were repeatedly shown to associate with different types of cancer, specifically with lung cancer.

It was shown that the level of SETD7 is decreased in lung cancer tissues compared to adjacent non-tumor tissues. Furthermore, downregulation of SETD7 lead to an increase in metastatic potential of non-small cell lung cancer (NSCLC) cells through elevated Twist and MMP2 expression [10]. Our studies also demonstrate the tumor suppressor effect of SETD7 in NSCLC: both knockout and knockdown of SETD7 led to increased proliferation and migration as well as elevated glycolysis levels via a c-Myc-dependent mechanism [11,12]. Importantly, according to our data, attenuation of SETD7 results in an increase in susceptibility of lung cancer cells to doxorubicin-induced genotoxic stress [11]. The tumor suppressive role of SETD7 methyltransferase in NSCLC was also confirmed in a recent study demonstrating the ability of SETD7 to methylate mutant KRAS protein and thereby to promote its degradation [13].

In addition to the regulation of proliferation, migration, and drug resistance of malignant cells, SETD7 was also shown to contribute to the orchestration of cancer-associated inflammation and immune response. It was demonstrated that methyltransferase SETD7 acts as a negative regulator of RelA stability and hinders expression of NFkB-dependent cytokines IL-6, IL-8, and CXCL10 (interferon gamma-induced protein 10, IP-10) in both human and murine cells [14]. In human hepatic cancer cells, SETD7 was shown to facilitate hepatitis C virus replication through downregulation of Interferon α-1 (*IFNA1*), Interferon β (*IFNB*), and IFN-stimulated genes (ISG) [15]. Mechanistically, this effect on interferon signaling was manifested due to SETD7-mediated inhibition of the nuclear translocation of IRF7 and NFkB. It was also shown that in bone marrow stem cells surrounded by an aging microenvironment, SETD7 expression is under negative regulation by IL-6. SETD7 decrease, in turn, led to inhibition of osteogenic differentiation and an increase in IL-1 and TNF-α production [16].

Along with evidence showing that SETD7 attenuates immune responses, several studies demonstrated that this KMT can also induce cytokine production and inflammatory processes. Thus, pharmacological inhibition of SETD7 by cyproheptadine and sinefungin suppressed the secretion of TNF-α, IL-6, and IL-8 by β-glucan-trained human macrophages [17]. Furthermore, the study performed on human bronchial epithelial cells revealed the ability of SETD7 to induce IL-6 and IL-8 production through an NF-κB-dependent mechanism [18].

Collectively, the data described above strongly imply SETD7 in the regulation of innate immunity factors, particularly those governed by the STING signaling cascade. The cGAS–STING signaling pathway is a key mechanism of innate immunity, specifically involved in the recognition of and defense against cytosolic double-stranded DNA (dsDNA) [19]. Upon binding to dsDNA, cGAS (cyclic GMP–AMP synthase) catalyzes the synthesis of 2′-3′-cyclic GMP-AMP (2′3′-cGAMP), a second messenger that activates the adaptor STING protein [20]. STING is an endoplasmic reticulum (ER)-resident protein that, upon binding to 2′3′-cGAMP, translocates to the ER–Golgi intermediate compartment (ERGIC)/Golgi, where it recruits and activates TBK1 kinase. TBK1 subsequently undergoes activation of trans-autophosphorylation and phosphorylates both STING and the transcription factor IRF3. Phosphorylation of IRF3 promotes its dimerization, nuclear translocation, and activation of type I interferon expression (IFIT1–3, MX1, OAS1, CXCL9–11) and interleukins (IL1β and IL6) via JAK/STAT- and NF-κB-dependent signaling pathways [21,22,23].

This evidence indicates that SETD7 plays a significant and versatile role in modulating inflammation and immune responses, in particular those associated with tumorigenesis. Despite the acknowledged significance of the immune pathways in the initiation and progression of malignant tumors, the effect of SETD7 in the context of cancer-associated immunity of lung adenocarcinoma (LUAD) has not been investigated to date. In this study we focus on the role of SETD7 in the regulation of inflammation-related signaling cascades, specifically, the STING-dependent molecular pathway.

## 2. Results

### 2.1. SETD7 Involved in Immune Profile Regulation in Lung Adenocarcinoma

**Figure 1:**
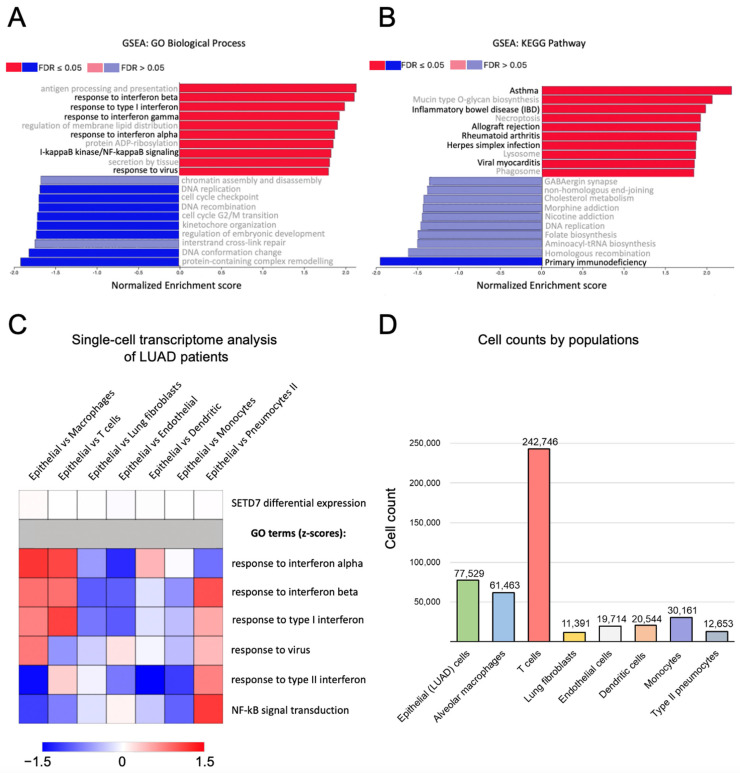
(**A**,**B**): Gene Set Enrichment Analysis (GSEA) of SETD7-associated biological processes (Gene Ontology, GO) (**A**) and pathways (Kyoto Encyclopedia of Genes and Genomes, KEGG) (**B**) in LUAD samples. Processes and pathways related to the immune response are shown in black, and all others are shown in gray. FDR-false discovery rate. (**C**,**D**): Analysis of single-cell RNA-seq data for differential expression of SETD7 and Gene Ontology terms in cell populations of LUAD samples. (**C**): Heatmap demonstrating SETD7 differential expression and scaled expression values (z-scores) of GO terms in epithelial cells relative to the indicated cell types. (**D**): Cell counts in analyzed cell populations.

To identify the potential role of SETD7 in the modulation of the immune status of LUAD cells, we performed Gene Set Enrichment Analysis (GSEA) using GO biological process and KEGG pathway enrichment analysis of SETD7-associated gene expression profiles. As a result, we revealed a strong and statistically significant positive correlation between SETD7 and the response of LUAD cells to type I (α and β) and type II (γ) interferons, as well as NF-κB signaling and antiviral responses (Figure 1A). Furthermore, the KEGG pathway analysis identified a positive correlation between SETD7 and processes that are tightly associated with immune system function or dysfunction, including asthma, inflammatory bowel disease, rheumatoid arthritis, allograft rejection, herpesvirus infection, and viral myocarditis, while a strong negative correlation was observed between SETD7 and primary immunodeficiency (Figure 1B). These findings indicate that SETD7 is a candidate regulator of the tumor-associated immune landscape in LUAD.

To further investigate the potential role of SETD7 in regulation of the immune response in LUAD cells, we analyzed publicly available single-cell RNA-seq data using the CZ CELLxGENE platform [24]. To this end, we integrated four datasets [25,26,27,28] to compare SETD7 expression and GO terms positively correlated with SETD7 in LUAD GSEA across both epithelial cells (that represent malignant cells) and the most abundant non-epithelial cell populations in LUAD single-cell RNA-seq datasets (Figure 1C,D).

According to the analysis, SETD7 expression shows low variability across the analyzed groups, including immune cell populations, lung fibroblasts, endothelial cells, and type II pneumocytes (Figure 1C). In parallel, analyzed GO terms demonstrate pronounced differential expression between cell populations. In particular, type I interferon-related genes show cell type specificity and display higher expression in the epithelial population compared to lung macrophages and T cells, while showing lower expression in type II pneumocytes, lung fibroblasts, and endothelial cells relative to the epithelial population (Figure 1C). Importantly, we observe a lack of substantial heterogeneity in SETD7 expression across the most abundant cell populations in LUAD tissue, whereas other GO term-associated genes vary considerably between populations and likely contribute to bulk RNA-seq data.

### 2.2. Altered SETD7 Expression Is Associated with Changes in Transcription of Inflammatory and Interferon Response Genes

**Figure 2:**
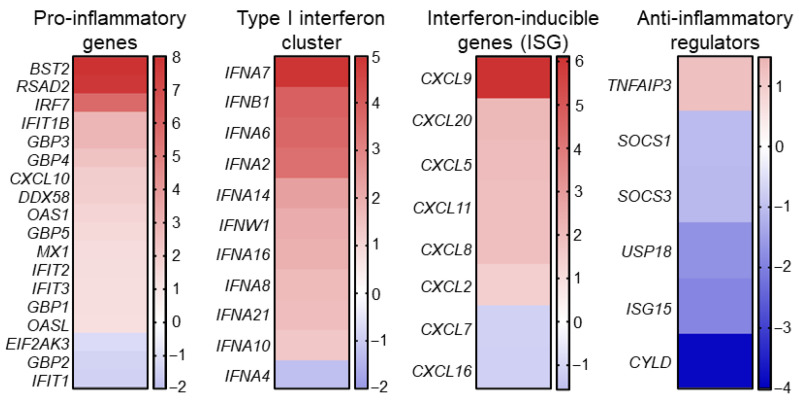
Differential expression of STING-associated inflammatory and interferon response genes following SETD7 knockdown in U2OS osteosarcoma cells. Heatmap displaying the log_2_ fold-change expression of 35 differentially expressed genes grouped into four functional clusters: pro-inflammatory genes, type I interferon cluster, interferon-inducible genes (ISG), and anti-inflammatory regulators, in SETD7 knockdown (KD) versus control U2OS cells as determined by microarray analysis. Color scales indicate relative up- or down-regulation of gene expression within each cluster. The heatmap was generated using GraphPad Prism 8 software.

To further investigate the potential role of SETD7 in the regulation of immune and inflammatory responses, we performed the analysis of microarray data previously obtained by our research group for U2OS human osteosarcoma cells with tetracycline-inducible SETD7 knockdown (SETD7 KD) and control U2OS pSuperior cells [12]. From this dataset, we extracted a subset of genes which are involved in pro-inflammatory and interferon signaling pathways and for which significant changes in expression in SETD7 KD compared to the control cell line were shown. The selected genes were grouped into four functional categories: (I) pro-inflammatory genes, (II) a type I interferon cluster, (III) interferon-inducible genes (ISGs), and (IV) anti-inflammatory regulators (Figure 2).

As a result, we revealed that the pro-inflammatory gene cluster shows increased expression of key markers of the innate immune response, including *BST2*, *RSAD2*, *IRF7*, *IFIT1-3*, *OAS1*, *MX1*, and *CXCL10*. Concurrently, the type I interferon cluster is characterized by upregulation of the majority of IFN family genes (including *IFNA2*, *IFNA6*, *IFNA7*, and *IFNB1*), indicating an enhanced basal interferon response with decreased SETD7 levels. A similar trend was also revealed for the ISG cluster represented by chemokines and mediators of cell migration (*CXCL9*, *CXCL11*, *CCL5*, *CCL20*): their expression is increased in SETD7 knockdown cells. Conversely, the expression of genes involved in negative regulation of inflammation and interferon signaling is predominantly decreased in response to SETD7 attenuation (Figure 2).

Thus, comparative expression analysis between SETD7 KD cells and control cells demonstrated a significant shift in the transcriptional profile towards activation of inflammatory and antiviral programs. Particularly, the observed upregulation of IFN family genes, the ISG cluster, and *IRF7* suggest a potential involvement of SETD7 in the regulation of STING-mediated immune activation.

Since the described microarray data were obtained in osteosarcoma cells, the effect of SETD7 on the innate immunity-specific expression profile required further analysis. To this end, we further analyzed the RNAseq dataset GSE229344 (GEO) obtained from adenovirus-infected H1299 cells. Comparison of SETD7 expression levels in control and infected cells revealed a statistically significant attenuation of SETD7 (~1.4-fold) upon viral transduction (Figure S1A), supporting a potential role of SETD7 in the regulation of innate immunity in cancer cells.

### 2.3. Salmon Sperm DNA (sspDNA) Transfection Protocol for STING Pathway Activation

Most non-genetic strategies for activating STING signaling in cell models involve the use of direct STING agonists, such as cyclic dinucleotides (CDNs; e.g., eukaryotic 2′3′-cGAMP or bacterial c-di-GMP, c-di-AMP, and 3′3′-cGAMP), small-molecule agonists (e.g., diABZI, MSA-2, or DMXAA for murine cell models), or transfection of short synthetic dsDNA molecules 40–100 bp [29,30,31,32,33,34]. To further investigate the role of SETD7 in STING signaling, we aimed to establish a cost-effective and reproducible DNA-mediated activation model using commercially available salmon sperm DNA (sspDNA).

#### 2.3.1. Salmon Sperm DNA Concentration Affects the Intensity of STING Signaling Activation

**Figure 3:**
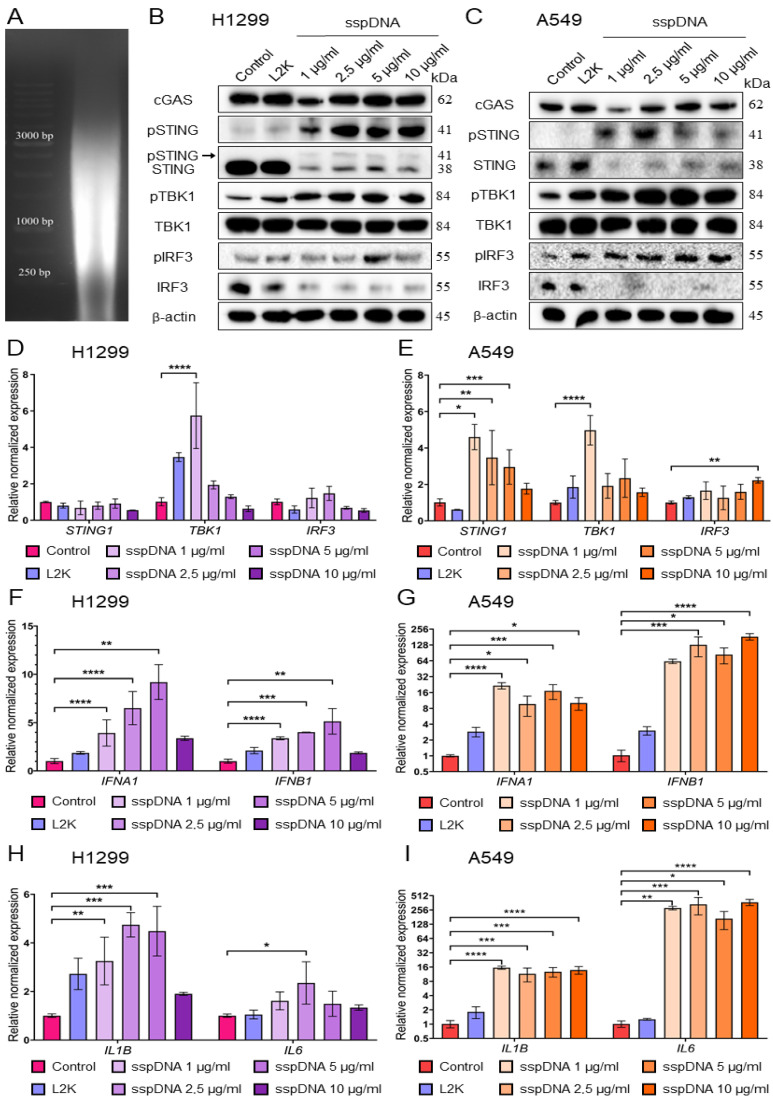
The analysis of the effect of salmon sperm DNA (sspDNA) concentration on STING pathway activation. (**A**) Agarose gel electrophoresis of the sspDNA used for transfection. DNA fragments ranging from ~250 to 3000 bp are shown with a 1 kb ladder. (**B**,**C**) Western blot analysis of cGAS and phosphorylated STING, TBK1, and IRF3 in H1299 (**B**) andA549 (**C**) cell lines treated with 1, 2.5, 5, and 10 µg/mL concentrations of sspDNA for 6 h. The black arrow indicates the phosphorylated form of STING detected by antibodies against total STING (**D**–**I**) qPCR analysis of the expression of *STING1*, *TBK1*, *IRF3* (**D**,**E**), IFNα-1 and IFNβ (*IFNA1, IFNB1*) (**F**,**G**) and interleukins 1β and 6 (*IL1B*, *IL6*) (**H**,**I**) in H1299 (**D**,**F**,**H**) and A549 (**E**,**G**,**I**) cell lines treated with corresponding concentrations of sspDNA for 6 h. Gene expression was normalized to *GAPDH*. Statistical analysis was performed using one-way ANOVA with *p* values defined as * *p* < 0.033 ** *p* < 0.0021, *** *p* < 0.0002, **** *p* < 0.0001.

First, we assessed the size distribution of DNA fragments in the sspDNA preparation (UltraPure™ Salmon Sperm DNA Solution, Invitrogen, Waltham, MA, USA). The material was used as supplied by the manufacturer without further fragmentation or enzymatic processing prior to analysis or experimental application. As shown in Figure 3A, the analyzed sspDNA predominantly contains fragments ranging from 250 to 3000 bp, corresponding to lengths previously reported to induce efficient activation of the cGAS–STING pathway (Figure 3A).

To determine the optimal concentration for cGAS–STING pathway activation, H1299 and A549 lung cancer cells were transfected with increasing concentrations of sspDNA for 6 h. Untreated cells and cells treated with the transfection reagent Lipofectamine 2000 (L2K) were used as negative controls.

According to Western blot analysis results, sspDNA transfection stimulated the increase in the levels of phosphorylated forms of STING, TBK1 and IRF3 proteins in both A549 and H1299 cell lines (Figure 3B,C and Figure S2A,B), consistent with activation of STING signaling in response to sspDNA transfection. The effect of sspDNA transfection differed between the two cell lines. In H1299, the most pronounced phosphorylation of the three analyzed proteins after 6 h incubation was observed at 5 µg/mL sspDNA, while 10 µg/mL resulted in a moderate decrease in pIRF3 levels (Figure 3B). In A549, the highest level of pSTING was detected at 2.5 µg/mL sspDNA, reducing as the concentration further increased. Phosphorylation of TBK1 and IRF3 increased starting from the minimum concentration (1 µg/mL) and remained stable within the 2.5–10 µg/mL range in A549 (Figure 3C). Importantly, the levels of STING, TBK1 and IRF3 proteins and their phosphorylated forms were significantly lower in A549 cells and required much longer exposure during chemiluminescent detection.

The effect of sspDNA transfection on the expression of *STING1*, *TBK1* and *IRF3* mRNA was also analyzed by qRT-PCR. Significant transfection-induced stimulation of *STING1* mRNA expression was observed only for A549 but not for the H1299 cell line (Figure 3D,E). Notably, *TBK1* expression was significantly elevated in both cell lines transfected with 1 µg/mL, followed by a gradual decline as the concentration increased (Figure 3D,E). *IRF3* mRNA did not show any elevation in either of the two cell models (Figure 3D,E).

At the mRNA level, the most informative indicators of STING cascade activation are the levels of IRF3 transcription targets—interferons *IFNA1/B1* and interleukins *IL1B* and *IL6*. Both cell lines exhibited an increase in mRNA levels of the analyzed interferons and interleukins; however, the effect was substantially more pronounced in A549 cells (Figure 3F–I). Thus, *IFNA1* and *IL1B* expression increased up to 7-fold in response to transfection with 1–10 µg/mL sspDNA compared to the L2K-treated control, while *IFNB1* and *IL6* mRNA levels demonstrated up to a 200-fold increase without decreasing at high sspDNA concentrations (Figure 3G). In H1299 cells we observed about a 4-fold increase compared to L2K treatment for *IFNA1* and about a 2-fold elevation for *IFNB1*, *IL1B* and *IL6* (Figure 3F,H). Furthermore, a decrease in cytokine expression was observed at the highest concentrations of sspDNA in H1299, which is consistent with the pIRF3 level (Figure 3B,D). Importantly, the expression levels of analyzed interferons and interleukins were lower in A549 cells compared to H1299 (Figure S1B).

On the basis of our data demonstrating both pronounced STING, TBK1 and IRF3 phosphorylation and induction of cytokine expression, and previous reports indicating that the A549 non-small cell lung cancer cell line exhibits attenuated STING signaling compared to H1299 [35], we selected H1299 cells for further experiments. In this cell line, 5 µg/mL sspDNA induced robust phosphorylation of STING, TBK1 and IRF3 and strong induction of STING-dependent cytokine genes. Increasing the concentration to 10 µg/mL did not further enhance pathway activation and was associated with a reduction in pIRF3 levels. Therefore, 5 µg/mL was selected as the optimal concentration for subsequent studies.

#### 2.3.2. Kinetics of STING Pathway Activation Following sspDNA Transfection

Based on the results described above, specifically biphasic changes in expression and phosphorylation levels of STING pathway components depending on the sspDNA concentration, we aimed to monitor the dynamics of STING activation upon sspDNA transfection at different time points.

**Figure 4:**
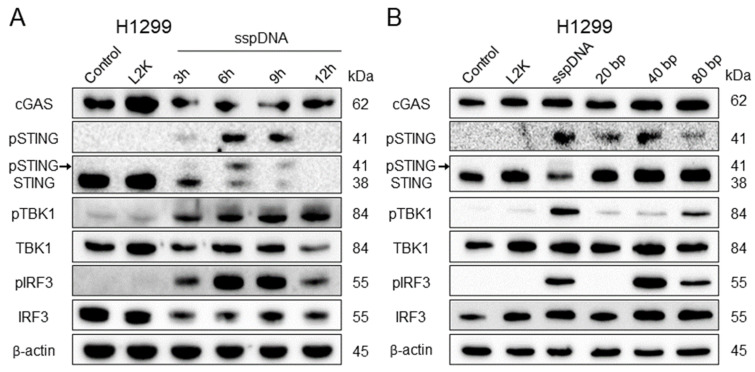
STING pathway activation in H1299 cells transfected with sspDNA and short synthetic oligonucleotides. (**A**) Time-dependent phosphorylation of STING signaling pathway proteins following transfection with sspDNA (5 µg/mL). (**B**) Phosphorylation of STING signaling pathway proteins after transfection with synthetic DNA fragments of different lengths. Cells were transfected with sspDNA or synthetic oligonucleotides (5 µg/mL) for 6 h. The black arrow indicates the phosphorylated form of STING detected by antibodies against total STING.

As shown in Figure 4A, the maximal levels of STING and IRF3 phosphorylation were observed at 6 and 9 h, while the phosphorylated form of TBK1 gradually increased with incubation time (Figure 4A and Figure S2C). Notably, a decrease in the non-phosphorylated form of STING was observed in parallel with pSTING accumulation in this and other experiments (Figure 4A). Incubation for 6 h most potently activated STING and IRF3 and resulted in a near-maximal increase in pTBK1. Accordingly, the 6 h time point was selected for subsequent STING signaling experiments.

#### 2.3.3. Comparison of STING Activation Induced by sspDNA and Short Oligonucleotides

As indicated above, transfection with synthetic DNA fragments of 40–100 bp is often used for activation of the STING-dependent immune response in vitro. However, this approach is highly sensitive to the length and concentration of dsDNA, which may be critical for routine application.

In order to perform a comparative analysis of the effectiveness of sspDNA and short oligonucleotides in the activation of STING signaling, we transfected H1299 cells with 5 µg/mL synthetic 20-, 40-, and 80 bp dsDNA molecules and 5 µg/mL sspDNA for 6 h. Untreated cells and cells treated with L2K were used as negative controls.

As shown in Figure 4B, 20 bp oligonucleotides induced only limited activation of STING without detectable phosphorylation of TBK1 and IRF3. Transfection with 40 bp dsDNA molecules promoted phosphorylation of STING and IRF3. In turn, 80 bp fragments caused substantial accumulation of pTBK1 and pIRF3, but not pSTING (Figure 4B and Figure S2D), while sspDNA transfection resulted in high levels of pSTING and pTBK1 and more coordinated activation of all three STING pathway components (Figure 4B). Thus, we demonstrated that transfection with sspDNA is not inferior in efficiency to the use of synthetic oligonucleotides and is an even more potent inducer of STING signaling in vitro.

### 2.4. The Role of SETD7 in the Regulation of STING Signaling and Innate Immune Response in the H1299 Cells

#### 2.4.1. SETD7 Affects STING Signaling-Dependent Expression of Pro-Inflammatory and Apoptose-Regulating Genes in NSCLC Cells in Response to sspDNA Transfection

To elucidate the role of SETD7 in the STING-dependent inflammatory response, we used H1299 cells with stable SETD7 knockdown (H1299 SETD7 KD) and H1299 cell expressing scrambled shRNA as a control (H1299 scr). Transfection with 5 µg/mL sspDNA for 6 h was performed as an optimal approach for the induction of the STING pathway.

**Figure 5:**
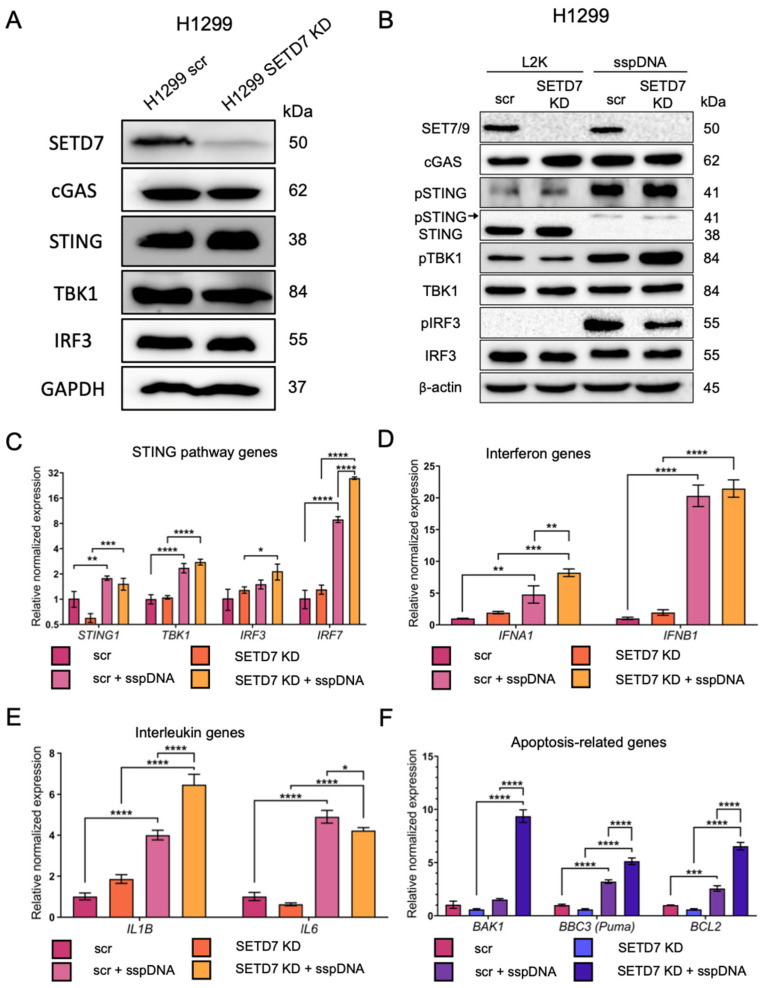
The analysis of the effect of sspDNA transfection on the SETD7 KD and scr H1299 cells. (**A**) Western blot analysis of basal levels of cGAS, STING, TBK1, and IRF3 proteins in H1299 SETD7 KD and scr H1299 cells. (**B**) Western blot analysis of cGAS and phosphorylated STING, TBK1, and IRF3 and their corresponding total protein levels in H1299 scr and SETD7 KD cells treated with 5 µg/mL sspDNA for 6 h. qPCR analysis of the expression of *STING1*, *TBK1*, *IRF3*, *IRF7.* The black arrow indicates the phosphorylated form of STING detected by antibodies against total STING (**C**), IFNα-1 and IFNβ (*IFNA1*, *IFNB1*) (**D**), interleukins 1β and 6 (*IL1B*, *IL6*) (**E**), and *BAK1*, *BBC3* (Puma) and *BCL2* (**F**) in H1299 scr and SETD7 KD cells. Gene expression was normalized to *GAPDH*. Statistical analysis was performed using one-way ANOVA with *p* values of * *p* < 0.033 ** *p* < 0.0021, *** *p* < 0.0002, and **** *p* < 0.0001.

Initially, we analyzed basal protein levels of STING, cGAS, TBK1, and IRF3 in H1299 cells with SETD7 knockdown. Western blot analysis did not reveal any significant changes in the non-phosphorylated forms of these STING pathway components under basal conditions (Figure 5A).

Next, we tested the effect of SETD7 knockdown on phosphorylation of STING, TBK1, and IRF3. According to the Western blot results, transfection with sspDNA stimulated accumulation of phosphorylated forms of STING, TBK1 and IRF3 in both cell lines (H1299 SETD7 KD and H1299 scr) compared to L2K-treated SETD7 KD and scr cells (Figure 5B and Figure S2E).

STING pathway activation in SETD7 KD and scr H1299 cells transfected with sspDNA shows that pSTING levels are almost equal in both H1299 scr and SETD7 KD cell lines, while pTBK1 and pIRF3 are increased and decreased, respectively, in H1299 SETD7 KD cells compared to H1299 scr cells, which corresponds to the observed asynchronous dynamics of IRF3 and TBK1 activation in the time-course experiments (Figure 4A).

Since SETD7 was shown to methylate both histone and non-histone targets, we tested whether there is any physical interaction between SETD7 and STING cascade proteins cGAS, STING, TBK1 and IRF3. We performed a GST pulldown assay using SETD7–GST purified protein and cell extracts prepared from H1299 cells. The eficiency of pulldown was validated by detection of known SETD7-interacting partners: histone H3 and the RNA-binding protein Sam68 (Figure S1C) [9]. We did not detect any interaction of SETD7 with STING pathway proteins or with the DNA-binding alarmin HMGB1, a known component of STING signaling (Figure S1C). This suggests that SETD7 likely regulates the cytokine response genes primarily at the mRNA level. Furthermore, STRING analysis of SETD7 interactome revealed neither the presence of proteins that belong to the STING cascade nor the *bona fide* innate immunity factors (Figure S1D). At the same time, enrichment analysis of the reactome pathway expectedly revealed the following groups of proteins that interact with SETD7, thereby validating the results of this analysis (Figure S1D): gene expression, generic transcription pathway, chromatin-modifying enzymes, and epigenetic regulation of gene expression.

Next, we analyzed the expression of *STING1*, *TBK1*, and *IRF3* mRNAs in response to 6 h sspDNA transfection, and additionally included *IRF7* in this analysis because it was significantly elevated in SETD7 KD U2OS cells according to microarray data (Figure 2). Untreated H1299 scr and SETD7 KD cell lines were used in this experiment as an internal control for effective STING pathway induction. As a result, we observed a moderate elevation of *IRF3* expression and no significant changes in the expression levels of *STING1* and *TBK1* (Figure 5B). Importantly, *IRF7* expression in H1299 SETD7 KD was increased by at least 3.5-fold compared to H1299 scr cells, which is consistent with the microarray data.

The qRT-PCR analysis of the effect of SETD7 on the type I interferons IFN-α (*IFNA1*) and IFN-β (*IFNB1*), and the interleukins *IL1B* and *IL6*, demonstrated that *IFNA1* and *IFNB1* mRNA levels were increased in the H1299 SETD7 KD cell line (Figure 5C,D). At the same time, attenuation of SETD7 led to a moderate decrease in *IL6* expression, while *IFNB1* remained unchanged (Figure 5C,D).

We further tested an effect of SETD7 knockdown on the expression of IRF3-dependent pro-apoptotic genes *BAK1* (BAK) and *BBC3* (PUMA), and the anti-apoptotic *BCL2* (Bcl-2). Intriguingly, we observed the elevation of all three of these factors in response to sspDNA transfection (Figure 5E). The most prominent enhancement was detected for *BAK1* mRNA, suggesting that SETD7 may contribute to IRF3-dependent apoptosis activation and the switching of STING signaling between pro-inflammatory and pro-apoptotic outputs.

To assess whether sspDNA transfection induces apoptotic cell death, we performed Annexin V staining in H1299 scramble and SETD7 knockdown cells treated with L2K or transfected with 5 µg/mL sspDNA at the 6 h time point. Cells treated with 5 µM camptothecin for 24 h were used as a positive control for apoptotic cell death, and the ones left untreated were used as a negative control. We did not observe any increase in apoptosis in response to sspDNA transfection, whereas L2K treatment moderately increased the proportion of Annexin V-positive cells in both cell lines (Figure S1F). This suggests that at least at this early time point, sspDNA-induced activation of pro-apoptotic gene expression does not lead to apoptosis, but instead initiates a transcriptional program associated with cell fate decisions.

Another important question was whether the p53 transcription factor may influence the ability of SETD7 to regulate apoptosis-related genes in response to sspDNA transfection. Since H1299 cells do not express p53, we used a genetically modified H1299 cell line with tetracycline-inducible expression of wild-type p53, previously described by our group [12,36]. Using this system, only BCL2 expression showed a statistically significant change in p53-expressing versus p53-null cells at the 6 h time point after transfection (Figure S1E). These data indicate that SETD7 can regulate the expression of pro-apoptotic genes in both p53-dependent and independent manners [1,37] and may also cooperate with p53 in suppressing the expression of the anti-apoptotic gene BCL2 in response to foreign DNA.

#### 2.4.2. STING Inhibitors H-151 and 4-PBA Abolish the Effect of SETD7 on the Expression of Cytokine and Apoptosis-Related Genes

**Figure 6:**
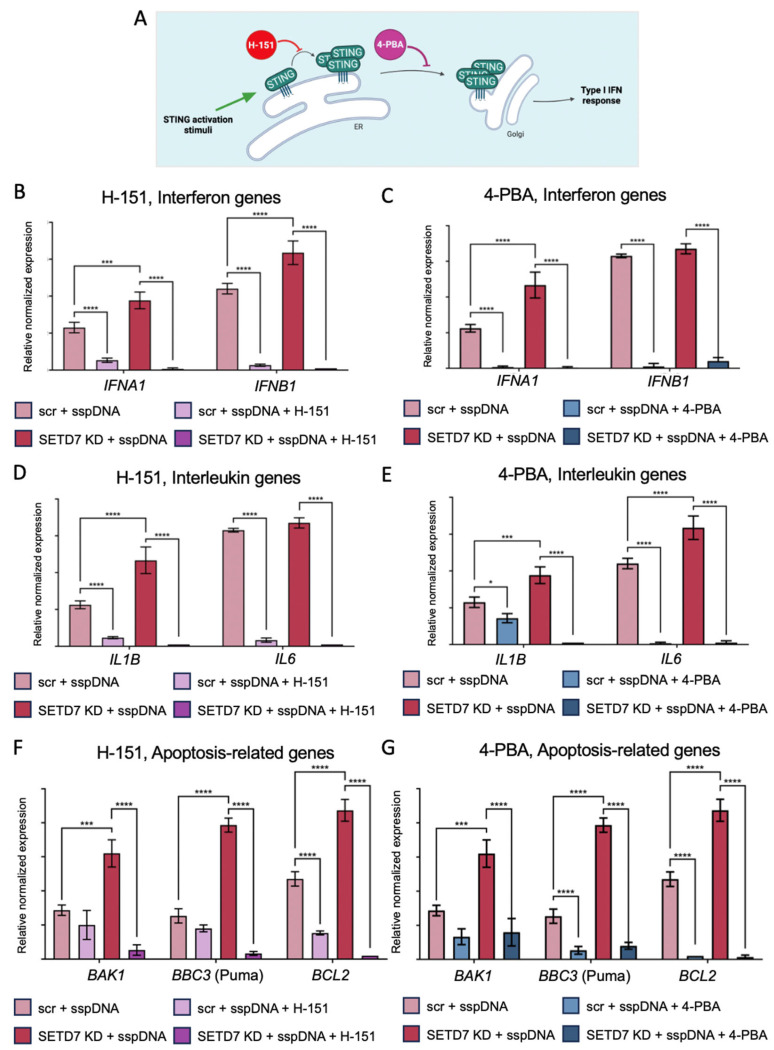
The effect of H-151 and 4-PBA on SETD7-mediated gene expression in response to sspDNA transfection in SETD7 KD and scr H1299 cells. (**A**): Scheme demonstrating the mechanisms of action of H-151 and 4-PBA on STING protein. qPCR analysis of the expression of IFNα-1 and IFNβ (*IFNA1*, *IFNB1*) (**B**,**C**), interleukins 1β and 6 (*IL1B*, *IL6*) (**D**,**E**), and *BAK1*, *BBC3* (Puma) and *BCL2* (**F**,**G**) in H1299 scr and SETD7 KD cells treated with 2 µM H-151 (**B**,**D**,**F**) or 10 mM 4-PBA (**C**,**E**,**G**) for 2 h prior to sspDNA transfection. Gene expression was normalized to *GAPDH*. Statistical analysis was performed using one-way ANOVA with *p* values of * *p* < 0.033, *** *p* < 0.0002, and **** *p* < 0.0001.

To confirm the STING-dependent effect of SETD7 on the expression of cytokine and apoptosis-related genes in response to sspDNA transfection, we used two STING inhibitors with distinct mechanisms of action. The selective small-molecule inhibitor H-151 targets the transmembrane domain of the STING protein and blocks its activating post-translational modification of palmitoylation, and hence assembly into an active multimeric complex [38] (Figure 6A), whereas 4-PBA (4-phenylbutyric acid) is an FDA-approved ER stress inhibitor that prevents STING trafficking from the ER to the ER–Golgi intermediate compartment (ERGIC) and suppresses STING signaling [39] (Figure 6A). Treatment with H-151 and 4-PBA (2 µM and 10 mM, respectively) was performed for 2 h prior to transfection with sspDNA. The expression of STING signaling components, type I interferon-, interleukin-, and apoptosis-related genes was measured by qRT-PCR at the 6 h time point (Figure 6B–G and Figure S1F).

Following treatment with both H-151 and 4-PBA, we observed a marked decrease in the expression of cytokines and, notably, apoptosis-related genes in both H1299 scr and SETD7 knockdown cell lines. Importantly, *IRF7* expression was decreased in a similar manner in both cell lines, which is consistent with the fact that *IRF7* is a transcriptional target of *IRF3*.

These data suggest that SETD7 transcriptional effects on cytokine and apoptosis-related gene expression in response to sspDNA transfection at least in part are STING-dependent and propose a mechanism for the regulation of cytokine responses in LUAD cells.

#### 2.4.3. SETD7 Regulates the Susceptibility of LUAD Cells to Viral Infection

**Figure 7:**
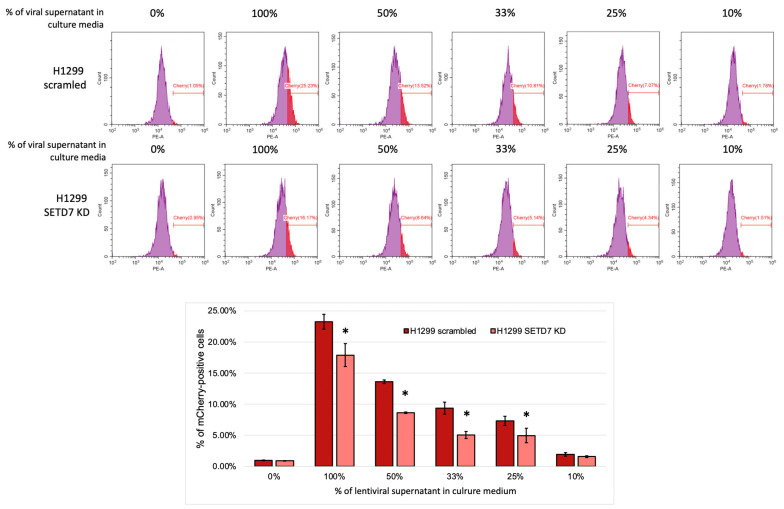
The impact of SETD7 on lentiviral infection in H1299 cells. Flow cytometry data demonstrating the percentage of mCherry-positive scr and SETD7 KD cells after 24 h of infection with indicated dilutions of viral supernatant. Statistical analysis was performed using one-way ANOVA with *p* values of * *p* < 0.05.

Our data indicate the involvement of SETD7 in the regulation of innate immune programs and STING signaling pathway-mediated cytokine responses. One of the key functional contexts for STING pathway activation is viral infection. To elucidate the biological significance of the proposed mechanism, we performed a viral infection assay using H1299 scr and SETD7 KD cells with lentiviral particles carrying a backbone vector encoding the mCherry fluorescent protein. A series of non-concentrated viral supernatant dilutions was used for 24 h incubation of H1299 cells. The efficiency of transduction was evaluated by flow cytometry. As a result, we demonstrated that SETD7 KD H1299 cells exhibited significantly lower transduction efficiency compared to control scr cells (Figure 7). This result further supports the effect of SETD7 on cGAS/STING-regulated pathways and suggests a potential role of SETD7 in fundamental cellular programs, such as antiviral defense.

## 3. Discussion

As a result of GSEA, we found a strong positive association between SETD7 expression and biological processes such as responses to type I and II interferons, NF-κB signaling, and antiviral responses in LUAD (Figure 1A), which indicates the potential role of this methyltransferase in the orchestration of the inflammatory and cytokine profiles of lung cancer cells. The analysis of KEGG pathways revealed a positive correlation between SETD7 and pathological states which are closely related to inflammatory and immune response processes, such as asthma, inflammatory bowel disease, rheumatoid arthritis, and allograft rejection (Figure 1B). Furthermore, the negative correlation with SETD7 was identified for the primary immunodeficiency state, which further confirms the supposed role of this methyltransferase in the immune profile of lung cancer cells.

Previous studies have reported that SETD7 regulates NF-κB transcriptional activity in both positive and negative manners depending on the cellular context and alters the expression of cytokines such as *IL6*, *IL1B*, and *CXCL10* [7,14,37,40].

Although the role of SETD7 in immune and inflammatory molecular cascades in lung cells has not been sufficiently investigated, the increased levels of inflammation markers *IL6*, *IFNG,* and *IL17A* in the colon cells of SETD7 knockout mice were reported [41]. This data is consistent with our microarray analysis results obtained for U2OS osteosarcoma cells with SETD7 knockdown (Figure 2). In this study, we demonstrated that attenuation of SETD7 leads to an increase in pro-inflammatory genes, the type I interferon cluster, and interferon-inducible genes, and at the same time a decrease in the expression of anti-inflammatory genes. Although this result was obtained in osteosarcoma cells, this extrapolative approach has proven productive in the past, as we were able to confirm microarray findings obtained from U2OS SETD7 KD cells in several LUAD cell lines, including H1299 and A549 [12]. Additionally, a bioinformatic analysis of an RNA-seq dataset capturing expression changes in H1299 cells infected with empty adenoviral particles revealed a significant decrease in SETD7 expression in virus-infected cells (Figure S1A). Undoubtedly, a comprehensive RNA-seq-based transcriptomic analysis will help clarify additional aspects of SETD7-mediated regulation of innate immunity in LUAD cells and will be the focus of our future studies.

As indicated above for NF-κB, SETD7-dependent regulation of gene expression may be controversial and depend on tissue context, which possibly explains the opposite effects of SETD7 on immune-related transcriptional profiles observed in LUAD and osteosarcoma cells (Figure 1 and Figure 2).

**Figure 8:**
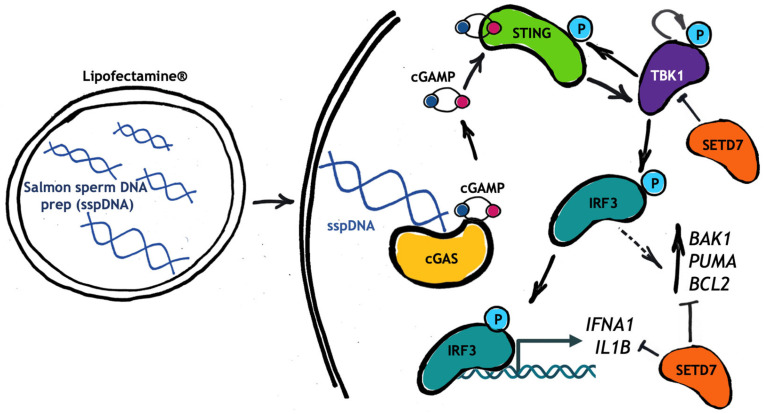
Schematic illustration demonstrating the effect of SETD7 on the STING signaling cascade and the expression of BCL-2 family members in response to sspDNA transfection.

Based on the RNA-seq and microarray data, we focused on the role of SETD7 in the STING-dependent innate immune signaling pathway and developed a reproducible, cost-effective, and time-efficient method for STING cascade activation. Using two LUAD cell lines, H1299 and A549, we demonstrated that transfection with 5 µg/mL of untreated commercially available salmon sperm DNA (sspDNA) preparation for 6 h efficiently induces phosphorylation of STING, TBK1 and IRF3, which in turn leads to enhanced expression of the pro-inflammatory IRF3-dependent cytokines type I interferons *IFNA1* and *IFNB1*, and the interleukins *IL1B* and *IL6* (Figure 8). We showed that untreated sspDNA is not inferior and even surpasses synthetic 20–80 bp oligonucleotides in STING cascade activation efficiency when used for transfection of H1299 cells (Figure 4B). Mechanistically, the greater efficacy of sspDNA preps compared with synthetic 20–80 bp oligonucleotides in activation of STING signaling is not unexpected. Previously published data convincingly demonstrate a positive correlation between DNA fragment length and the efficiency of cGAS activation, cGAMP synthesis, and induction of IFN gene expression [34,42]. Luecke et al. showed that the highest level of cGAS activation and cytokine production were achieved with the 4003 bp fragments, compared to 94 bp and 500 bp fragments. Additionally, there is evidence that cGAS is sensitive to the structural flexibility of DNA fragments [43,44], which may give whole-genome DNA preparations a functional advantage over synthetic DNA oligonucleotides [34]. Indeed, the nucleotide preparations used in most publications, including our own study, consist of randomly generated sequences that do not account for optimal DNA flexibility. Supporting our hypothesis, both the STING-specific inhibitor H151 and a blocker of the STING translocation to the ERGIC, 4-PBA, markedly reduced the transcriptional response induced by sspDNA (Figure 6), indicating that the observed effects are likely mediated via the cGAS-STING pathway.

Notably, we detected moderate activation of STING signaling pathway in response to L2K treatment in both H1299 and A549 cells (Figure 3B,C and Figure 5B). We consider that the observed activation of STING cascade proteins is associated with an increased rate of apoptosis in cells treated with L2K (Figure S1F). These data are consistent with the results of other studies [45,46]. Since STING signaling is sensitive to the presence of dsDNA, including that released from dead cells, induction of the STING pathway by L2K, at least in part, may be associated with the cytotoxicity of the transfection reagent. In line with this, L2K has been shown to induce ER stress, autophagy, xenobiotic stress, and ROS response, all of which can modulate the STING signaling cascade [47,48].

According to our results, A549 cells demonstrated decreased phosphorylation levels of STING, TBK1 and IRF3 compared to H1299 cells in response to sspDNA transfection, together with reduced basal expression of pro-inflammatory cytokines. This observation corresponds to previously published data demonstrating attenuated STING signaling in the A549 LUAD cell line [49,50,51]. However, we observed significantly stronger induction of *IFNA1/B1*, *IL1B* and *IL6* expression in response to sspDNA transfection in A549. It has been demonstrated that foreign DNA may be recognized by cGAS-independent pathways and induce a type I interferon response. The RIG-1 receptor was shown to identify RNA products of RNA polymerase III generated on cytosolic dsDNA templates, which leads to pro-inflammatory cytokine expression [52]. Later studies have shown that RIG-1 induces formation of the MAVS signalosome, which in turn activates NF-κB-dependent pro-inflammatory cytokine expression [53]. Furthermore, the dsDNA-mediated induction of the AIM2 inflammasome may contribute to *IL1B* expression independently of cGAS-STING signaling [54]. Both of these cytokine-inducing pathways have been reported to be pronounced in A549 LUAD cells [55,56,57]. In order to focus on the STING-dependent immune response in our study, we selected the H1299 cell line for further analysis of the effect of SETD7 on the STING pathway in LUAD cells.

Investigation into the role of SETD7 in STING signaling activation in LUAD cells in vitro revealed increased expression of *IFNA1* and *IL1B* in SETD7-deficient H1299 cells, whereas *IFNB1* and *IL6* levels did not differ from those in H1299 cells with intact SETD7 expression (Figure 5C,D and Figure 6). The discrepancy between the results of the functional experiment and the data obtained using LUAD patients’ transcriptomic datasets may reflect several factors. First, the publicly available RNA-seq data obtained from patient biopsy material contain bulk tumor transcriptomes, including malignant, immune, and tumor microenvironment cells. It is known that LUAD tumors frequently display increased immune infiltration and interferon response signatures [58,59], which may influence correlations observed in bulk transcriptomic datasets. This hypothesis is supported by our single-cell RNA-seq bioinformatics analysis of LUAD samples, which revealed a substantial proportion of non-epithelial cell populations and pronounced heterogeneity in immune-related GO terms, alongside relatively low variability in SETD7 expression across these populations (Figure 1C,D). Second, as noted above, the effect of SETD7 is highly context-dependent, which has been observed for its ambivalent effects on NF-κB transcriptional activity and cytokine production. The H1299 cell line is a model of p53-null, EGFRwt, and NRASmut LUAD, which limits the ability to generalize the data obtained in these cells to all lung cancer models. Thus, we suggest that expanding the range of cell models will be necessary for future clarification of the role of SETD7 in the STING pathway in LUAD. Finally, future experiments on co-culture *in vitro* and *in vivo* should clarify whether SETD7 is a target of pro-inflammatory signaling cascades. This may explain the positive correlation between SETD7 and immune signaling cascades detected in the RNA-seq analysis.

Notably, when analyzing the effect of sspDNA transfection in H1299 cells with attenuated SETD7, we observed increased expression of the pro-apoptotic members of the BCL-2 family BAK (*BAK1*) and PUMA (*BBC3*), as well as the anti-apoptotic member Bcl-2 (*BCL2*) (Figure 5E and Figure 6). It has been repeatedly shown that the STING cascade is known not only to induce interferon signaling but also to promote mitochondrial apoptosis through IRF3-dependent activation of pro-apoptotic proteins such as BAX and NOXA at both protein and mRNA levels [44,60]. SETD7 is a known positive regulator of the p53 transcription factor [6], and hence we were interested whether these two proteins may cooperate in the regulation of apoptosis-related genes in response to sspDNA transfection. Surprisingly, ectopic expression of wild-type p53 in the absence of DNA damage did not affect BAK1 and PUMA, but influenced only BCL2 expression (Figure S1E), suggesting that regulation of these two pro-apoptotic genes by SETD7 is largely independent of p53 in the context of STING activation and innate immune signaling.

Our results demonstrate the elevation of both pro-apoptotic and pro-survival members of the BCL-2 family in SETD7 knockdown cells in response to sspDNA transfection, which may indicate the role of SETD7 in regulating cell fate decisions (Figure 8). It has been repeatedly shown that BCL-2 family members are regulated by common transcription factors such as p53, MYC, and E2F1 [61,62,63], which allows for fine-tuning of the balance between pro-survival and pro-death signals. This concept of the regulation and functioning of BCL-2 family proteins is known as the BCL-2 rheostat and has been described for various cellular responses including immune response [64,65,66].

Recently, increasing attention has been paid to the role of SETD7 in immune signaling and inflammation-related pathological conditions [67]. To date, the mechanism controlling the switch of STING signaling between pro-inflammatory and pro-apoptotic outcomes remains unclear, and our findings suggest that SETD7 may represent a potential regulator of this balance.

## 4. Materials and Methods

### 4.1. GSEA

Gene Set Enrichment Analysis (GSEA) of the dataset of lung adenocarcinoma (LUAD) patients (National Cancer Institute’s Clinical Proteomic Tumor Analysis Consortium (CPTAC) pan-cancer LUAD) was performed to identify the association between SETD7 expression and GO biological processes and KEGG pathways. The LinkIntepreter tool of the LinkedOmics suite was used for the analysis [68]. Pearson’s correlation test was used as the statistical method.

### 4.2. Microarray Data Analysis

To reveal the potential SETD7 effects on inflammation-related signaling cascades, we analyzed microarray data obtained by our research group for U2OS cells with Tet-inducible SETD7 knockdown and control U2OS pSuperior cells with unaltered SETD7 expression. The raw data are available at Mendeley Datasets Repository (https://data.mendeley.com/datasets/hkgfsz9yhn/1, accessed on 10 May 2021) [12]. We analyzed fold change in the expression of key participants of inflammation-related signaling cascades in SETD7 KD vs. SETD wt U2OS cells.

### 4.3. Cell Cultures

Human LUAD cell lines H1299 and A549 were purchased from ATCC and maintained in RPMI 1640 and Dulbecco’s Modified Eagle Medium correspondingly. The growth medium was supplemented with 10% fetal bovine serum (FBS) (Gibco, Waltham, MA, USA) and gentamicin (50 μg/mL). The establishment of the H1299 cell line with the SETD7 knockdown (H1299 SETD7 KD) and control cell line (H1299 scr), as well as H1299 with tetracycline-inducible p53wt expression, was described previously [11,12].

### 4.4. DNA and Transfection

UltraPure™ salmon sperm DNA solution (Invitrogen, Carlsbad, CA, USA) (sspDNA) was used for STING signaling activation. The size of DNA molecules was analyzed using agarose gel electrophoresis. The specifically sized (20, 40, and 80 bp) DNA molecules used in the experiments were as follows:

80 bp Forward–TAGAGAATGGGGTGTACGAATAAGGAAGGGGGGGGTGTGGTTGGAAGTCTGGAATGGGGTGATGGAATAAGGAATCTAGG;
80 bp Reverse–CCTAGATTCCTTATTCCATCACCCCATTCCAGACTTCCAACCACACCCCCCCCTTCCTTATTCGTACACCCCATTCTCTA;
40 bp Forward–AGAGAATGGGGTGTACGAATAAGGAAGGGGGGGGTCTAGG;
40 bp Reverse–CCTAGACCCCCCCCTTCCTTATTCGTACACCCCATTCTCT;
20 bp Forward–GACCTCAGACCTACCTCAGC;
20 bp Reverse–GCTGAGGTAGGTCTGAGGTC.

DNA transfection was performed using Lipofectamine™ 2000 Transfection Reagent (Invitrogen, Waltham, MA, USA) (L2K) according to the manufacturer’s recommendations. Specifically, 20 µL of Lipofectamine was added to 500 µL of Opti-MEM medium (Gibco, Waltham, MA, USA) and kept for 5 min at room temperature. In parallel, the required amount of DNA was diluted in 500 µL of Opti-MEM and left for 5 min at room temperature. The two solutions were then combined and maintained to form complexes for 25 min at room temperature. The mixture was added to H1299 or A549 cells in Opti-MEM (70% confluency), followed by incubation at 37 °C with 5% CO_2_.

For the 3 h time point, cells were collected after 3 h of exposure to the transfection mixture. For the 6, 9, and 12 h time points, cells were exposed to the transfection mixture for 4 h, followed by incubation in complete medium for 2, 5, and 8 h, respectively. The following final concentrations of DNA were used: 1 µg/mL, 2.5 µg/mL, 5 µg/mL and 10 µg/mL for sspDNA and 5 µg/mL for 80 bp, 40 bp and 20 bp oligonucleotides.

### 4.5. Quantitative RT-PCR (qRT-PCR)

Total RNA was extracted from cells using ExtractRNA solution (Evrogen, Moscow, Russia) according to the manufacturer’s recommendations. To avoid genomic DNA contamination the isolated RNA was treated with DNAse E (Evrogen, Moscow, Russia). RNA 1 µg of RNA was used for reverse transcription using the MMLV RT kit (Evrogen, Moscow, Russia), which was then used as a template for the qPCRmix-HS SYBR kit (Evrogen, Moscow, Russia). The mRNA expression levels were calculated relative to GAPDH house-keeping control by the ΔΔCt method. A one-way analysis of variance (ANOVA) was conducted to examine the statistical difference between studied groups. The assumption of normality was assessed using the Shapiro–Wilk test, and homogeneity of variances was verified using Bartlett’s test. The sequences of primers used are given in Supplementary Table S1.

### 4.6. Western Blot Analysis

For Western blot analysis, whole-cell extracts were prepared using radioimmunoprecipitation assay (RIPA) buffer. For SDS-PAGE, Laemmli buffer was added to the cell extracts, followed by denaturation at 97 °C for 5 min. After SDS-PAGE, proteins were transferred to a PVDF membrane using the Trans-Blot Turbo system (Bio-Rad, Hercules, CA, USA). Protein transfer was performed in Tris–glycine buffer (TGB) containing 20% ethanol for 1.5 h at 100 V and 4 °C. The following primary antibodies were used: anti-β-actin (Sigma-Aldrich, St. Louis, MO, USA), anti-GAPDH, anti-cGAS, anti-STING, anti-p-STING (Ser366), anti-TBK1, anti-p-TBK1 (Ser172), anti-SETD7 (Cell Signaling, Danvers, MA, USA), anti-IRF3, and anti-p-IRF3 (Ser396) (Affinity, Ancaster, ON, Canada). Secondary antibodies included anti-mouse and anti-rabbit (1:10,000; Sigma-Aldrich, St. Louis, MO, USA). Chemiluminescent signals were detected using Clarity™ Western ECL Substrate (Bio-Rad, Hercules, CA, USA) and a ChemiDoc MP Imaging System (Bio-Rad, Hercules, CA, USA). Densitometric analysis was performed using Image Lab software version 5.0 (Bio-Rad, Hercules, CA, USA) relative to loading controls (β-actin or GAPDH).

### 4.7. GST Pulldown Assay

The GST pulldown assay was performed as previously described [69]. SETD7–GST protein was purified using affinity chromatography on Glutathione Sepharose 4B (Sigma-Aldrich, St. Louis, MO, USA) from *E. coli* transformed with pGEX-5X-1 vector (GE Healthcare, Chicago, IL, USA) containing the SETD7 insert [9]. The appropriate amount of GST was purified and used as a negative control. Cell lysates were prepared from H1299 cells and used for incubation with SETD7–GST and GST proteins immobilized on Glutathione Sepharose for 3 h with rotation at +4 °C. After washing with PBS, the bound proteins were eluted and analyzed by Western blot.

### 4.8. The Bioinformatic Analysis of RNAseq Data

The analysis of SETD7 participation in biological processes and pathways in LUAD samples was performed using the GSEA enrichment method via the LinkedOmics tool [10]. The RNA-seq dataset GSE229344 was analyzed using the Phantasus 1.31.1 tool. Single-cell RNAseq analysis was performed using the CZ CELLxGENE tool [24]. A combined analysis of four datasets [25,26,27,28] was performed. Differential expression (DE) was calculated for SETD7. The expression of genes included in GO terms (response_to_interferon_alpha; response_to_interferon_beta; response_to_type_I_interferon; response_to_type_II_interferon; CANONICAL_NF_KAPPAB_SIGNAL_TRANSDUCTION; response_to_virus) was calculated using Seurat (AddModuleScore). Expression differences between the epithelial cell population and other populations were calculated and z-score normalized for heatmap visualization.

### 4.9. Annexin V Staining

Detection of Annexin V-positive apoptotic cells was performed using the FITC Annexin V Apoptosis Detection Kit (BD Pharmingen, San Diego, CA, USA) according to the manufacturer’s instructions. 7-Aminoactinomycin D (7-AAD) was used as a viability dye. Flow cytometry analysis was performed using a CytoFLEX flow cytometer (Beckman Coulter, Brea, CA, USA) to evaluate FITC- and 7-AAD-positive signals.

### 4.10. Viral Infection Assay

For the viral particle production, plasmids pUltraHot-mCherry-BLM-WT (Addgene, Watertown, MA, USA, #206966), pMD2.G, and psPAX2 were co-transfected into HEK293T cells using TurboFect transfection reagent (Thermo Fisher Scientific, Waltham, MA, USA) to generate mCherry-expressing lentiviral particles. The viral supernatant was harvested 48 h after transfection, filtered through a 0.45 µm PES filter, and diluted with complete RPMI 1640 medium in the indicated proportions. The diluted viral supernatants were added to H1299 scr and H1299 SETD7 KD cells seeded in 24-well plates the day before at a density of 1 × 10^5^ cells per well. After 24 h of incubation, cells were analyzed using a CytoFLEX flow cytometer (Beckman Coulter, Brea, CA, USA) to evaluate the proportion of mCherry-positive cells.

## 5. Conclusions

This study focuses on the role of the SETD7 lysine methyltransferase in the regulation of the STING-dependent innate immune response in human LUAD cells. A reproducible and cost-effective method for STING pathway activation using an untreated commercial preparation of salmon sperm DNA was developed and may be applied to a wide range of research tasks related to the investigation of the STING cascade. Importantly, we demonstrated the complex role of SETD7 in the regulation of the IRF3-dependent transcriptional program. Further investigation of the crosstalk between SETD7 and STING pathway components may clarify the possibility of controlling the switch of this cascade from inflammation to apoptosis, which may be relevant for future medical applications and drug development.

## Data Availability

Microarray data obtained by our research group for U2OS cells with SETD7 knockdown and control U2OS pSuperior cells is available in the public domain: https://data.mendeley.com/datasets/hkgfsz9yhn/1, accessed on 10 May 2021. The original contributions presented in this study are included in the article/Supplementary Materials. Further inquiries can be directed to the corresponding authors.

## Supplementary Materials

The supporting information can be downloaded at https://www.mdpi.com/article/10.3390/ijms27094020/s1.

## Abbreviations

The following abbreviations are used in this manuscript:

4-PBA	4-phenylbutyric acid
ANOVA	A one-way analysis of variance
CPTAC	National Cancer Institute’s Clinical Proteomic Tumor Analysis Consortium
DE	Differential expression
dsDNA	Double-stranded DNA
ERGIC	Endoplasmic Reticulum–Golgi Intermediate Compartment
GSEA	Gene Set Enrichment Analysis
H3K4	position 4 in histone H3
IFN	Interferon
IFNA1	Interferon α-1
IFNB	Interferon β
ISG	Interferon-stimulated genes
KEGG	Kyoto Encyclopedia of Genes and Genomes
KMT	Lysine-specific methyltransferase
L2K	Lipofectamine 2000
LUAD	Lung Adenocarcinoma
NSCLC	non-small cell lung cancer
qRT-PCR	Quantitative Reverse Transcription Polymerase Chain Reaction
RIPA	Radioimmunoprecipitation assay buffer
sspDNA	Salmon sperm DNA
TGB	Tris-glycine buffer
