# Supplementary material for: Methyltransferase SETD7 as a Regulator of STING-Dependent Cytokine Response in Lung Cancer Cells
Source: Int J Mol Sci. 2026 Apr 30;27(9):4020. doi: 10.3390/ijms27094020 (PMC13163566; doi:10.3390/ijms27094020)
Supplement: Supplementary file 1 [file ijms-27-04020-s001.zip › Figure S1 .pdf]

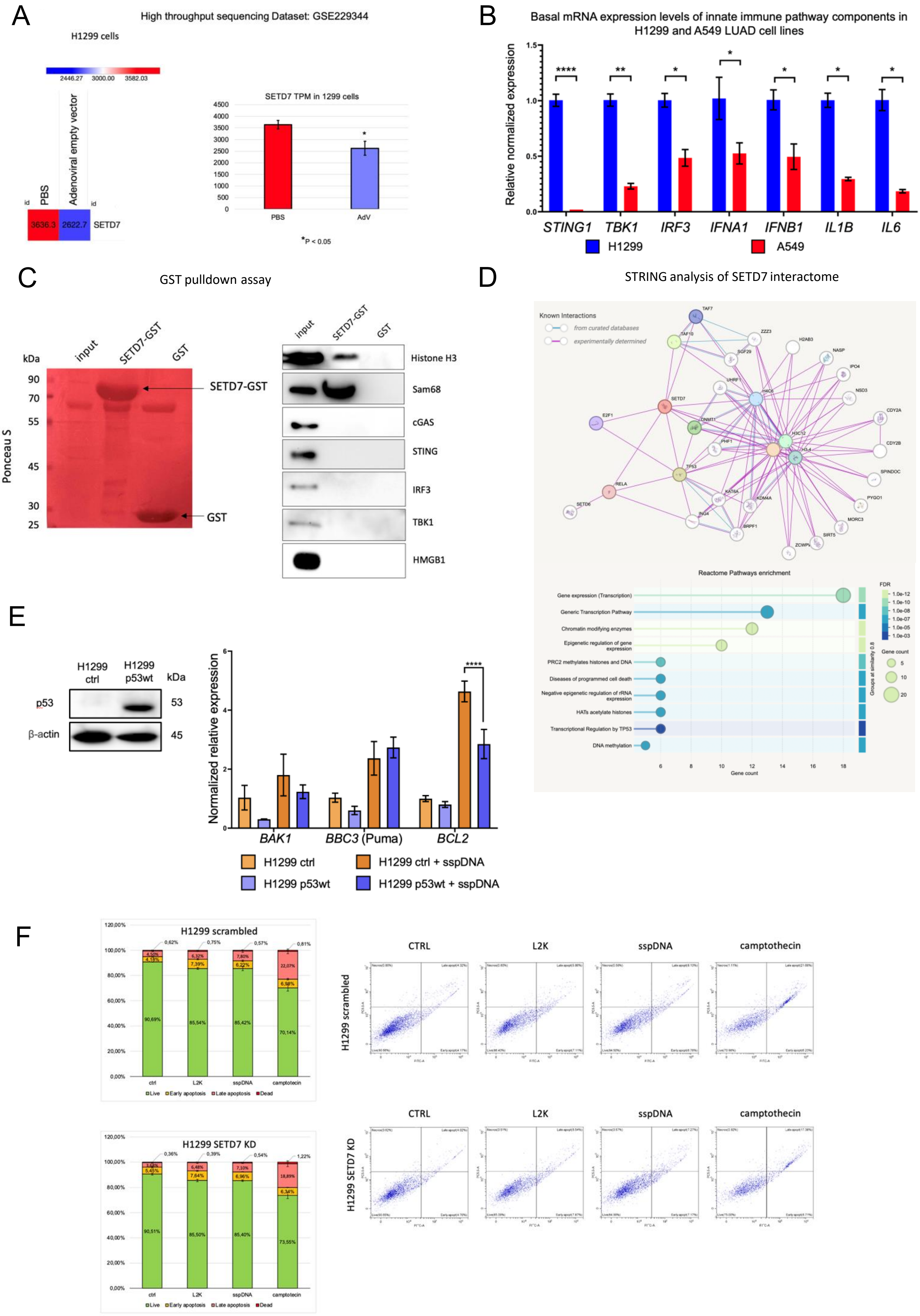

Supplementary Figure S1 (S1). (A) The analysis of SETD7 expression levels in H1299 cells infected with empty adenoviral particles. (B) Basal mRNA expression levels of innate immune pathway components in H1299 and A549 LUAD cell lines. The expression levels of STING1, TBK1, IRF3, IFNA1, IFNB1, IL1B, and IL6 were standardized against GAPDH. Statistical analysis was performed using one-way ANOVA with p values being *p < 0.033 **p < 0.0021, ***p < 0.0002, ****p < 0.0001. (C) GST pulldown assay using purified SETD7-GST protein and cell extracts from H1299 cells. Ponceau S staining shows the purified SETD7-GST and GST proteins used in the assay. Interacting proteins were detected by Western blot. (D) The analysis of SETD7 interactome using STRING database (http://string-db.org). (E) mRNA expression levels of BAK1, BBC3 (PUMA), and BCL2 in H1299 tet-on control and tet-on p53wt cells at the 6 h time point after transfection with sspDNA. 1 µg/ml doxycycline was aaded to the culture medium 48 h before the experiment. Statistical analysis was performed using one-way ANOVA, ****p < 0.0001. (F) Flow cytometric analysis of apoptosis in H1299 scr and SETD7 KD cells. The effects of L2K treatment and sspDNA transfection on apoptosis levels were analyzed at the 6 h time point. Treatment with 5 µM camptothecin for 24 h was used as a positive control.
