# Supplementary material for: Methyltransferase SETD7 as a Regulator of STING-Dependent Cytokine Response in Lung Cancer Cells
Source: Int J Mol Sci. 2026 Apr 30;27(9):4020. doi: 10.3390/ijms27094020 (PMC13163566; doi:10.3390/ijms27094020)
Supplement: Supplementary file 1 [file ijms-27-04020-s001.zip › Figure S3.pdf]

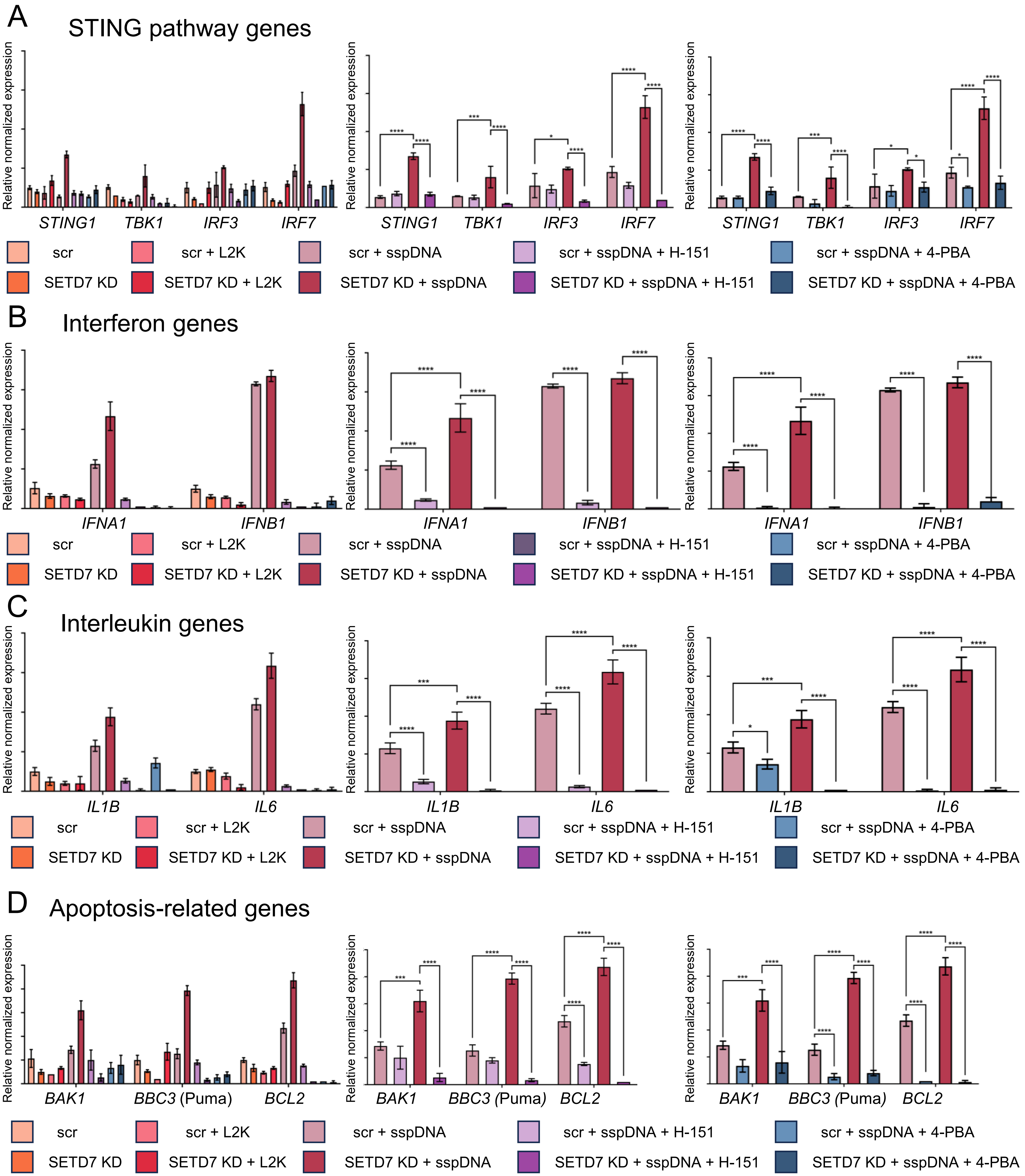

Figure S3 (S3). The effects of H-151 and 4-PBA on SETD7-mediated gene expression in response to sspDNA transfection in SETD7 KD and scr H1299 cells (expanded panel) at the 6 h time point. qPCR analysis of the expression of STING1, TBK1, IRF3, and IRF7 (**A**); IFNA1 and IFNB1 (B); IL1B and IL6 (C); and BAK1, BBC3 (PUMA), and BCL2 (D) in H1299 scr and SETD7 KD cells treated with 2 µM H-151 or 10 mM 4-PBA for 2 h before transfection. The analysis was performed at the 6 h time point. Untreated and L2K-treated cells were used as controls. Statistical analysis was performed using one-way ANOVA with p values being *p < 0.033 **p < 0.0021, ***p < 0.0002, ****p < 0.0001.
