# Supplementary material for: Methyltransferase SETD7 as a Regulator of STING-Dependent Cytokine Response in Lung Cancer Cells
Source: Int J Mol Sci. 2026 Apr 30;27(9):4020. doi: 10.3390/ijms27094020 (PMC13163566; doi:10.3390/ijms27094020)
Supplement: Supplementary file 1 [file ijms-27-04020-s001.zip › Supplementary table S1.pdf]

cGAS	Forward	CACGAAGCCAAGACCTCCG
	Reverse	GTCGCACTTCAGTCTGAGCA
IFNA1	Forward	CTCATACACCAGGTCACGCT
	Reverse	GCAGGGGTGAGAGTCTTTGAA
IFNB1	Forward	ACGCCGCATTGACCATCTAT
	Reverse	GTCTCATTCCAGGTCACGCT
IL1B	Forward	ATCCCCAGCCCTTTTGTTGA
	Reverse	TTTCTGTCAGGCGGGCTTTA
IL6	Forward	GACCCAACCACAAATGCCAG
	Reverse	GTGCCCATGCTACATTTGCC
IRF3	Forward	CACATTTCCAACAGCCACCC
	Reverse	GTAGGCCTTGTACTGGTCGG
IRF7	Forward	GCTGGACGTGACCATCATGTA
	Reverse	GGGCCGTATAGGAACGTGC
STING	Forward	CCTGAGTCTCAGAACAACTGCC
	Reverse	GGTCTTCAAGCTGCCCACAGTA
TBK1	Forward	TGATCTTTGGAGCATTGGGGT
	Reverse	AGGGTCTAAATGGCAGTGATCC
BAX	Forward	CAGCTCTGAGCAGATCATGAAGACA
	Reverse	GCCCATCTTCTTCCAGATGGTGAGC
BCL2	Forward	CCGCATCAGGGAAGGCTAGAG
	Reverse	CTGGGACACAGGCAGGTTCT
BBC3	Forward	GCGAGACTGTGGCCTTGTGT
	Reverse	CGTTCCAGGGTCCACCAAAGT
GAPDH	Forward	GTCTCCTCTGACTTCAACAGCG
	Reverse	ACCACCCTGTTGCTGTAGCCAA

Supplementary Table S1. Primers used for qRT-PCR
