# Supplementary figures and images for: Methyltransferase SETD7 as a Regulator of STING-Dependent Cytokine Response in Lung Cancer Cells
Source: Int J Mol Sci. 2026 Apr 30;27(9):4020. doi: 10.3390/ijms27094020 (PMC13163566; doi:10.3390/ijms27094020)

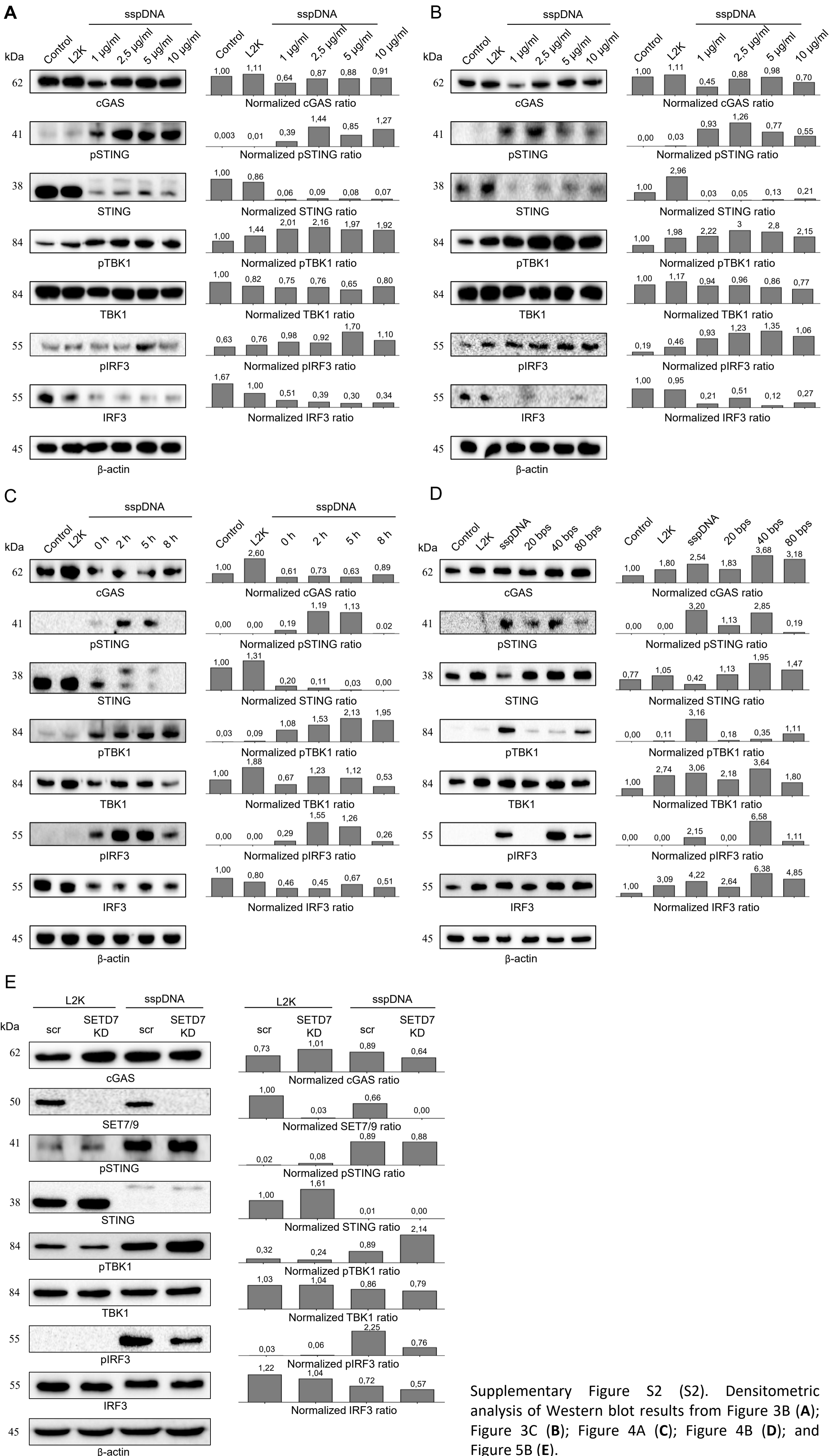

Supplement: Supplementary file 1 [file ijms-27-04020-s001.zip › Figure S2.pdf]
